# Relationship between atherosclerosis and occlusal support of natural teeth with mediating effect of atheroprotective nutrients: From the SONIC study

**DOI:** 10.1371/journal.pone.0182563

**Published:** 2017-08-17

**Authors:** Sayaka Tada, Kazunori Ikebe, Kei Kamide, Yasuyuki Gondo, Chisato Inomata, Hajime Takeshita, Ken-ich Matsuda, Masahiro Kitamura, Shinya Murakami, Mai Kabayama, Ryousuke Oguro, Chikako Nakama, Tatsuo Kawai, Koichi Yamamoto, Ken Sugimoto, Ayumi Shintani, Takuma Ishihara, Yasumichi Arai, Yukie Masui, Ryutaro Takahashi, Hiromi Rakugi, Yoshinobu Maeda

**Affiliations:** 1 Department of Oral Health Science, Division of Preventive Dentistry, Niigata University Graduate School of Medical and Dental Science, Niigata, Niigata, Japan; 2 Department of Prosthodontics, Gerodontology and Oral Rehabilitation, Osaka University Graduate School of Dentistry, Suita, Osaka, Japan; 3 Division of Health Science, Osaka University Graduate School of Medicine, Suita, Osaka, Japan; 4 Department of Geriatric and General Medicine, Osaka University Graduate School of Medicine, Suita, Osaka, Japan; 5 Department of Clinical Thanatology and Geriatric Behavioral Science, Osaka University Graduate School of Human Sciences, Suita, Osaka, Japan; 6 Department of Periodontology, Division of Oral Biology and Disease Control, Osaka University Graduate School of Dentistry, Suita, Osaka, Japan; 7 Department of Medical Statistics, Osaka City University Graduate School of Medicine, Osaka, Osaka, Japan; 8 School of Medicine, Keio University, Shinjuku-ku, Tokyo, Japan; 9 Tokyo Metropolitan Geriatric Hospital and Institute of Gerontology, Itabashi-ku, Tokyo, Japan; Leibniz-Institut fur Pflanzengenetik und Kulturpflanzenforschung Gatersleben, GERMANY

## Abstract

Whereas most of studies investigating relationship between oral health and atherosclerosis have focused on periodontitis, very few of them were examined about occlusal status of natural teeth which possibly influence dietary habit. The primary aim of this cross-sectional study was to investigate the association between the occlusal support of posterior teeth and the prevalence of atherosclerosis in community-dwelling septuagenarians. Also, the second aim was to test the hypothesis that the intake of key nutrients for atherosclerosis prevention would have a mediating effect on the relationship between the occlusal status and atherosclerosis. The study population included 468 community-dwelling dentate persons aged 69–71 years recruited from the local residential registration in Japan. Participants were divided into three groups, according to the number of occlusal support zones (OSZ) in the posterior area: Complete (four OSZ), Moderate (three or two OSZ), and Collapsed (one or no OSZ). Dietary intakes were assessed using a brief-type self-administered diet history questionnaire. Atherosclerosis was defined as carotid intima-media thickness ≧1.10 mm by using carotid ultrasonography test. The logistic or linear regression model was used in multivariate analysis to assess relationship between occlusal status and atherosclerosis, and the mediating effect of key nutrients within the relationship. Multivariable analysis showed a significant association between occlusal status and atherosclerosis (odds ratio for Collapsed group to Complete group: 1.87; 95% CI: 1.45–2.41), independent of periodontal status (odds ratio: 2.01, 95%CI: 1.46–2.78). Fish and shellfish, vitamin B6 and n-3PUFAs were significantly related to both of occlusal status and atherosclerosis, and also was indicated a mediating effect on the association between occlusal status and atherosclerosis. This study implied that, within the limitation of the cross-sectional study design, the reduced posterior occlusion was related to the increased prevalence of atherosclerosis via the decline of key dietary intakes among Japanese community-dwelling dentate individuals.

## Introduction

In older adults, oral health has a high impact on diet and nutrition, which can affect systemic health. In particular, a decline in masticatory function resulting from loss of natural occlusion in the posterior region is reported to make chewing difficult for older adults and to lead to avoidance of hard-to-chew foods such as vegetables, fruits, fish and shellfish [1–5]. On the other hand, there is increasing evidence that a higher intake of vegetables, fruits, fish and shellfish can reduce the risk of cardiovascular or cerebrovascular disease via favorable effects on disease contributors [6–12]. Thus, maintaining good occlusal support of natural teeth could possibly help prevent cardiovascular and cerebrovascular accidents, which are the most common causes of sudden death and disability in older adults worldwide.

Since the beginning of the 21st century, the relationship between atherosclerosis, which contributes to cardiovascular and cerebrovascular accidents, and periodontal disease has been the subject of growing research [13]. Recent literature reviews report a potential link between atherosclerosis and oral health through the inflammatory effects of periodontal disease [14,15].

Therefore, two pathways connecting oral health with atherosclerosis can be hypothesized: (a) the effect of bad dietary habits resulting from poor occlusal condition [1–12] and (b) the effect of inflammation associated with chronic periodontal infection on the circulatory system [13–15]. However, no previous studies have investigated the association between occlusal condition and atherosclerosis adjusting periodontal disease in same population.

The aim of this cross-sectional study was to investigate the relationship between the prevalence of atherosclerosis and both of occlusal and periodontal status in a community-dwelling septuagenarian. And secondly, it was also aimed to test a mediating effect of the intake of atheroprotective nutrients within the relation between the occlusal status and atherosclerosis. The null hypothesis of this study was that poor occlusal condition was not significantly associated with neither atherosclerosis nor atheroprotective nutrient intakes.

## Materials and methods

### Study population

This cross-sectional study was conducted as a baseline assessment for the prospective study of health and longevity called the SONIC (Septuagenarians, Octogenarians, Nonagenarians Investigation with Centenarians) Study. Participants were 495 community-dwelling septuagenarians aged 69–71 years who were recruited from the Basic Resident Registration of Itami (urban area) and Asago (rural area) in Hyogo Prefecture, Japan. Data collection was conducted in 2010 and 2011 at each local community hall.

For this study, 23 edentulous participants were excluded. In addition, four participants who did not attend the medical examination were excluded. Thus, 468 participants (Group A) were included in the multivariable assessment between occlusal status and atherosclerosis. Additionally, in analyzing of nutrient intakes, we excluded participants who reported either an extremely high energy intake (≥ 4000 kcal/d) or an extremely low energy intake (<600 kcal/d), who were receiving dietary counseling from a doctor or dietician at enrollment, or those with intentional dietary change during the preceding year. The final pool of participants for the analysis of nutrients numbered 371 (Group B).

The study protocol was approved by the Institutional Review Board of Osaka University Graduate Schools of Dentistry (approval number H22-E9) and Medicine (approval number 266). All participants gave written informed consent to participate.

### Dental examination

Oral examinations, including periodontal and dental examinations, were performed by registered dental clinicians. Occlusal support condition was categorized into one of three groups, based on the number of occlusal support zones (OSZ) which consist of occlusal contacts of natural teeth or fixed prosthesis on both side of the premolar and molar regions, which is basically from the concept of Eichner’s Index [16]: Complete (four OSZ: Eichner A1, A2 and A3), Moderate (three or two OSZ: Eichner B1 and B2), and Collapsed (one or no OSZ: Eichner B3, B4, C1 and C2). Periodontal condition was assessed by measuring periodontal pocket depth (PPD). All remaining teeth of participants were measured. If the probing depths varied within a site, the deepest reading obtained at that site was recorded; only one reading was recorded per site measurement. Periodontal condition was categorized into one of three groups, according to the participant’s deepest PPD: Healthy (PPD 3 mm or less), Moderate (PPD 4 or 5 mm), and Severe (PPD 6 mm or more).

### Evaluation of carotid atherosclerosis

Atherosclerosis was diagnosed with carotid ultrasonography. The left and right common carotid arteries were examined with a high-resolution duplex scanner (GE LOGIQ book XP; GE Healthcare, Tokyo, Japan), using a probe at a frequency of 7.5 MHz for the B-scan. Participants were examined in the supine position, with the head slightly turned away from the side being scanned. Following the same methodology as a previous study [17], the thickness of the carotid intima-media (IMT) and plaque were measured; the maximum was defined as the maximum IMT (max-IMT). In this study, atherosclerosis was defined as max-IMT ≥ 1.10, which is the value generally used for individuals in their seventies.

### Other medical examinations and diagnostic criteria

Blood samples were collected after overnight fasting. Levels of total cholesterol, high-density lipoprotein cholesterol (HDL-C), triglycerides (TG), and fasting plasma glucose were determined with biochemical testing. Low-density lipoprotein cholesterol (LDL-C) was calculated with the Friedwald formula. Dyslipidemia was defined as LDL-C ≥ 140 mg/dL, TG ≥ 150 mg/dL, HDL-C < 40 mg/dL, and/or medications for dyslipidemia [18]. Diabetes mellitus was defined as fasting plasma glucose ≥ 126 mg/dL and/or hemoglobin A_1c_ ≥ 6.5% and/or current medications for diabetes mellitus [19]. Systolic blood pressure (SBP) and diastolic blood pressure (DBP) were measured twice in a sitting position at the venue, using a calibrated standard mercury sphygmomanometer. Two measured BP levels were averaged. Hypertension was defined as SBP ≥ 140 mm Hg and/or DBP ≥ 90 mm Hg and/or current medications for hypertension [18,20].

### Questionnaire assessment

Socioeconomic and other factors were assessed based on answers to a questionnaire. Residential region was categorized as urban (Itami, population 7875/km^2^) or rural (Asago, population 81/km^2^). Education level was categorized into one of three groups, according to the number of years of education: <10 years, 10 to 12 years, and >12 years. Financial status was scaled with self-reported subjective assessment as follows: dissatisfied, moderately satisfied, and satisfied. Research staff questioned participants on their history of smoking and alcohol intake; responses were classified as never, past, or current.

### Nutrient intakes

Nutrient intakes during the preceding month were assessed using a brief-type self-administered diet history questionnaire (BDHQ) that measures consumption frequencies of selected food commonly consumed and calculates energy-adjusted dietary intakes. The methods used to calculate dietary intake and the validity of the BDHQ have been detailed in previous reports [21–23]. Values were indicated as a mean amount per 1000 kcal of energy to minimize the influence of dietary misreporting. Key nutrients for preventing atherosclerotic disease such as green and yellow vegetables, other vegetables, fruits, and fish and shellfish, and specifically dietary fiber, vitamin A, C, E, B6, B12, folic acid, n-3 polyunsaturated fatty acid (n-3PUFAs) were determined in this present study [24–29].

### Statistical methods

In assessing the association between the prevalence of atherosclerosis and each variable in the group A (n = 468), the outcome assessed in this study was set as the prevalence of atherosclerosis, and two predictors were defined as occlusal status and periodontal status. The other potential confounders were region of residence, education level, financial status, and risk factors for atherosclerosis, including gender, smoking habits, drinking habits, obesity (body mass index [BMI] ≥ 25), hypertension, diabetes mellitus, dyslipidemia, and hyperuricemia. The chi-square test was used in bivariate analysis to assess the association between the prevalence of atherosclerosis and each variable. Multiple logistic regression analysis was performed to examine the association between the prevalence of atherosclerosis and occlusal support and periodontal condition, adjusted for gender, region of residence, smoking habits, drinking habits, education level, diabetes mellitus, hypertension, dyslipidemia, and hyperuricemia. P-values less than 0.05 were considered to be statistically significant.

Secondly, among group B (n = 371), to determine between the occlusal status and the difference of means of each key dietary intake, the one-way ANOVA test was used. For the post hoc test, Tukey’s HSD test was used. To examine the relationship between each key dietary intake and both of atherosclerosis and occlusal status, multivariable linear regression model was used, adjusted for periodontal condition, gender, region of residence, smoking habits, drinking habits, education level, diabetes mellitus, hypertension, dyslipidemia, and hyperuricemia. *P*-values less than 0.10 were considered to be statistically significant.

And finally, to test the mediating effect of dietary intakes within the relation between occlusal status and atherosclerosis, multivariable logistic regression analysis was also performed.

Multiple imputations were conducted for missing data in multivariate analysis. Data were analyzed with PASW Statistics 18 software (formerly SPSS; IBM Company, Tokyo, Japan) and R software, Version 3.1.1 (10 July 2014, R Core Team, http://www.r-project.org/).

## Results

Overall of group A (n = 468), 217 participants (46%) were diagnosed as having carotid atherosclerosis. In terms of posterior occlusal support, 57% (n = 265) of participants had complete occlusion (four OSZ), 17% (n = 79) had moderate occlusion (three or two OSZ), and 26% (n = 117) had collapsed occlusion (one or no OSZ). Sixteen percent (n = 73) of all participants had healthy periodontal status, 40% (n = 185) had moderate periodontal disease, and 44% (n = 206) had severe periodontal disease. The statistical correlation between occlusal support and periodontal condition was very weak (Cramer’s V = .114, *p* = .017).

Bivariate analysis showed that occlusal support as well as periodontal condition was significantly associated with the prevalence of atherosclerosis. The associations between atherosclerosis and other potential confounders are shown in Table 1.

**Table 1:** Demographic and clinical characteristics of the group A by prevalence of atherosclerosis.

Variable ^a^	N	Atherosclerosis		*p*-value (χ^2^ test)
		Absence (n = 251)	Prevalence (n = 217)	
Occlusal support				0.002
Complete	265	155 (58%)	110 (42%)	
Moderate	79	46 (58%)	33 (42%)	
Collapsed	121	48 (40%)	73 (60%)	
Periodontal condition				0.006
Healthy	73	50 (68%)	23 (32%)	
Moderate	185	102 (55%)	83 (45%)	
Severe	206	97 (47%)	109 (53%)	
Gender				<0.001
Female	251	169 (67%)	82 (33%)	
Male	217	82 (38%)	135 (62%)	
Living region				0.091
Urban	237	118 (50%)	119 (50%)	
Country	231	133 (58%)	98 (42%)	
Self-rated financial status				0.816
Dissatisfied	130	67 (52%)	63 (48%)	
Moderate	233	128 (55%)	108 (45%)	
Satisfied	102	54 (53%)	48 (47%)	
Education level				0.021
≤9 years	110	51 (46%)	59 (54%)	
10–12 years	235	141 (60%)	94 (40%)	
≥13 years	121	58 (48%)	63 (52%)	
Smoking				<0.001
Never	274	176 (64%)	98 (36%)	
Past	131	49 (37%)	82 (63%)	
Current	39	8 (21%)	31 (79%)	
Alcohol exposure				<0.001
No	244	151 (62%)	93 (38%)	
Yes	203	86 (45%)	117 (55%)	
Hypertension				0.483
No	144	81 (56%)	63 (44%)	
Yes	323	169 (52%)	154 (48%)	
Diabetes mellitus				<0.001
No	313	185 (59%)	128 (41%)	
Yes	71	24 (34%)	47 (66%)	
Dyslipidemia				0.315
No	168	85 (51%)	83 (49%)	
Yes	263	146 (56%)	117 (44%)	
Hyperuricemia				0.016
No	375	209 (56%)	166 (44%)	
Yes	37	13 (35%)	24 (65%)	
Obesity				0.417
BMI<25	349	191 (55%)	158 (45%)	
BMI≥25	119	60 (50%)	59 (50%)	

a: Data are shown with counts and column percentages, i.e., N (%).

BMI: Body mass index

Table 2 shows the results of multivariable logistic regression analysis to examine the association between the prevalence of atherosclerosis and both occlusal status and periodontal condition, after adjustment for potential confounders, including gender, region of residence, smoking habits, drinking habits, education level, diabetes mellitus, hypertension, dyslipidemia, and hyperuricemia. Global testing indicated that this model predicts the prevalence of carotid atherosclerosis based on a group of independent variables (*p* < .001). The Collapsed group had significantly a higher risk of atherosclerosis (odds ratio (OR): 1.87, *p* = .013), compared with the Complete group; a reduction in the number of OSZ was significantly associated with the prevalence of atherosclerosis (*p* = .035). Individuals with severe periodontal disease were also at significantly higher risk of atherosclerosis than those with healthy periodontal tissues (OR: 2.01, *p* = .032).

**Table 2:** Odds ratios of main variables with adjustment for periodontal disease.

Variable	Odds ratio	95% CI	*p*-value
Occlusal support			
Complete	1.00		
Moderate	0.99	0.56, 1.75	0.958
Collapsed	1.87	1.14, 3.07	0.013
Periodontal condition			
Healthy	1.00		
Moderate	1.83	0.98, 3.44	0.059
Severe	2.01	1.06, 3.79	0.032
Gender			
Male	1.00		
Female	0.45	0.24, 0.83	0.011
Living region			
Urban	1.00		
Country	0.69	0.45, 1.07	0.100
Smoking			
Never	1.00		
Past	1.80	0.97, 3.36	0.063
Current	3.22	1.28, 8.11	0.013
Alcohol exposure			
No	1.00		
Yes	0.84	0.49, 1.44	0.520
Education level			
≤9 years	1.00		
10–12 years	0.57	0.34, 0.98	0.041
≥13 years	0.83	0.45, 1.51	0.533
Diabetes mellitus			
No	1.00		
Yes	2.45	1.42, 4.21	0.001
Hypertension			
No	1.00		
Yes	0.92	0.59, 1.43	0.703
Hyperuricemia			
No	1.00		
Yes	1.43	0.64, 3.20	0.385
Dyslipidemia			
No	1.00		
Yes	0.95	0.61, 1.48	0.817

As characteristics of the group B was indicated almost the same as the group A (Table 3), the group B was treated as a representative cohort of the group A in this study.

**Table 3:** Demographic and clinical characteristics of the group B by prevalence of atherosclerosis.

Variable ^a^	N	Atherosclerosis		*p*-value (χ^2^ test)
		Absence (n = 202)	Prevalence (n = 169)	
Occlusal support				0.051
Complete	223	129 (58%)	94 (42%)	
Moderate	56	33 (59%)	23 (41%)	
Collapsed	92	40 (43%)	52 (57%)	
Periodontal condition				0.053
Healthy	61	41 (67%)	20 (33%)	
Moderate	151	83 (55%)	68 (45%)	
Severe	159	78 (49%)	81 (51%)	
Gender				<0.001
Female	201	138 (69%)	63 (31%)	
Male	170	64 (38%)	106 (62%)	
Living region				0.218
Urban	189	97 (51%)	92 (49%)	
Country	182	105 (58%)	77 (42%)	
Self-rated financial status				0.758
Dissatisfied	108	56 (52%)	52 (48%)	
Moderate	176	99 (56%)	77 (44%)	
Satisfied	86	46 (53%)	40 (47%)	
Education level				0.234
≤9 years	86	43 (50%)	43 (50%)	
10–12 years	193	113 (59%)	80 (41%)	
≥13 years	91	45 (49%)	46 (51%)	
Smoking				<0.001
Never	227	146 (64%)	81 (36%)	
Past	96	37 (39%)	59 (61%)	
Current	33	6 (18%)	27 (82%)	
Alcohol exposure				<0.001
No	198	124 (63%)	74 (37%)	
Yes	162	69 (43%)	93 (57%)	
Hypertension				0.915
No	115	62 (54%)	53 (46%)	
Yes	255	139 (55%)	116 (45%)	
Diabetes mellitus				0.001
No	253	150 (59%)	103 (41%)	
Yes	46	15 (33%)	31 (67%)	
Dyslipidemia				0.399
No	139	71 (51%)	68 (49%)	
Yes	201	112 (56%)	89 (44%)	
Hyperuricemia				0.024
No	298	168 (56%)	130 (44%)	
Yes	29	10 (34%)	19 (66%)	
Obesity				0.186
BMI<25	284	160 (56%)	124 (44%)	
BMI≥25	87	42 (48%)	45 (52%)	

a: Data are shown with counts and column percentages, i.e., N (%)

The association between the occlusal status and the key dietary intakes was shown in Tables 4 and 5. From bivariate analysis (Table 4), all of key dietary intakes declined in Collapsed group. Although no significant differences of any key dietary intakes between Complete and Moderate groups were found, of Collapsed group, intakes of green and yellow vegetables, fish and shellfish, vitamin E, B6 and n-3PUFAs were significantly lower than those of Complete group.

**Table 4:** Unadjusted association between key food or nutrient intakes and occlusal support.

**Food Intakes**	**Occlusal Support**	**Mean**	**95% CI**	***p*-value**
Green and Yellow Vegetables (g/1000kcal)	Complete	71.5	66.6, 76.4	(Ref.)
	Moderate	68.5	57.7, 79.3	0.849
	Collapsed	59.4	64.2, 71.8	0.023
Other Vegetables (g/1000kcal)	Complete	106.8	100.0, 113.5	(Ref.)
	Moderate	101.4	89.1, 113.6	0.746
	Collapsed	97.6	87.7, 107.5	0.294
Fruits (g/1000kcal)	Complete	81.5	73.4, 89.5	(Ref.)
	Moderate	90.9	72.8, 108.9	0.575
	Collapsed	78.7	65.3, 92.1	0.933
Fish and Shellfish (g/1000kcal)	Complete	57.7	53.4, 62.0	(Ref.)
	Moderate	56.2	46.9, 65.6	0.945
	Collapsed	46.5	42.0, 51.0	0.009
**Nutrient Intakes**				
Vitamin A (μg retinol equivalent/ 1000kcal)	Complete	2.62	2.45, 2.79	(Ref.)
	Moderate	2.44	2.07, 2.81	0.621
	Collapsed	2.30	2.03, 2.55	0.095
Vitamin C (mg/1000kcal)	Complete	76.8	72.6, 81.1	(Ref.)
	Moderate	76.7	67.3, 86.0	1.000
	Collapsed	71.9	65.5, 78.3	0.438
Vitamin E (mg/1000kcal)	Complete	4.32	4.18, 4.47	(Ref.)
	Moderate	4.21	3.91, 4.50	0.743
	Collapsed	3.97	3.75, 4.20	0.025
Vitamin B6 (mg/1000kcal)	Complete	0.76	0.73, 0.78	(Ref.)
	Moderate	0.75	0.70, 0.80	0.970
	Collapsed	0.70	0.67, 0.74	0.048
Vitamin B12 (mg/1000kcal)	Complete	6.47	6.03, 6.92	(Ref.)
	Moderate	6.56	5.59, 7.54	0.983
	Collapsed	5.62	5.12, 6.12	0.078
Dietary Fibre (g/1000kcal)	Complete	7.24	6.98, 7.50	(Ref.)
	Moderate	7.27	6.65, 7.88	0.996
	Collapsed	6.94	6.49, 7.39	0.478
Follic Acid (μg/1000kcal)	Complete	221.9	209.6, 228.5	(Ref.)
	Moderate	208.4	188.4, 228.5	0.574
	Collapsed	203.1	189.6, 216.5	0.160
n-3PUFAs (g/1000kcal)	Complete	1.60	1.53, 1.67	(Ref.)
	Moderate	1.57	1.42, 1.72	0.919
	Collapsed	1.42	1.33, 1.51	0.013

CI: confidence interval, n-3PUFAs: n-3 polyunsaturated fatty acid

**Table 5:** Relationship of each key food or nutrient to atherosclerosis and to occlusal support, adjusted for covariates.

Food or Nutrients	< Atherosclerosis>		< Occlusal Support >	
	β1 (95% CI)	P-value	β2 (95% CI)	P-value
Green and Yellow Vegetables (g/1000kcal)	-5.02 (-13.1, -3.06)	0.223	-7.36 (-16.0, 1.30)	0.096
Other Vegetables (g/1000kcal)	-2.10 (-12.4, 8.22)	0.690	-5.54 (-16.65, 5.57)	0.328
Fruits (g/1000kcal)	-1.88 (-15.1, 11.3)	0.779	2.67 (-11.75, 17.10)	0.716
Fish and Shellfish (g/1000kcal)	-5.93 (-12.7, 0.83)	0.085	-9.57 (-16.86, -2.27)	0.010
Dietary Fiber (g/1000kcal)	-0.29 (-0.78, 0.19)	0.322	-0.09 (-0.53, 0.36)	0.706
Vitamin A (μg retinol equivalent/ 1000kcal)	-285.8 (-590.2, 18.6)	0.442	-191.7 (-487.7, 104.3)	0.204
Vitamin C (mg/1000kcal)	-4.77 (-12.3, 2.80)	0.770	-1.69 (-8.79, 5.42)	0.640
Vitamin E (mg/1000kcal)	-0.33 (-0.58, -0.07)	0.028	-0.20 (-0.45, 0.04)	0.107
Vitamin B6 (mg/1000kcal)	-0.05 (-0.09, -0.01)	0.033	-0.04 (-0.08, 0.004)	0.074
Vitamin B12 (mg/1000kcal)	-0.90 (-1.65, -0.15)	0.119	-0.82(-1.59, -0.05)	0.037
Folic Acid (μg/1000kcal)	-13.5 (-30.0, 3.02)	0.330	-7.37 (-23.0, 8.26)	0.355
N-3PUFAs (g/1000kcal)	-0.18 (-0.30, -0.06)	0.014	-0.16 (-0.28, -0.04)	0.012

β1 and β2: regression coefficient, CI: confidence interval, n-3PUFAs: n-3 polyunsaturated fatty acid. All models were adjusted for periodontal disease, gender, region of residence, smoking habits, drinking habits, education level, diabetes mellitus, hypertension, dyslipidemia, and hyperuricemia.

After adjusting confounders, green and yellow vegetables (*p* = .096), fish and shellfish (*p* = .010), vitamin B6 (*p* = .074), vitamin B12 (*p* = .037) and n-3PUFAs (*p* = .012) were significantly related to occlusal status (Complete & Moderate groups vs. Severe group) (Table 5). In terms of atherosclerosis, fish and shellfish (*p* = .085), vitamin E (*p* = .028), vitamin B6 (*p* = .033) and n-3PUFAs (*p* = .014) were significantly related (Table 5). Therefore, fish and shellfish, vitamin B6 and n-3PUFAs were the commonly related factors between occlusal status and atherosclerosis.

**Fig 1:**
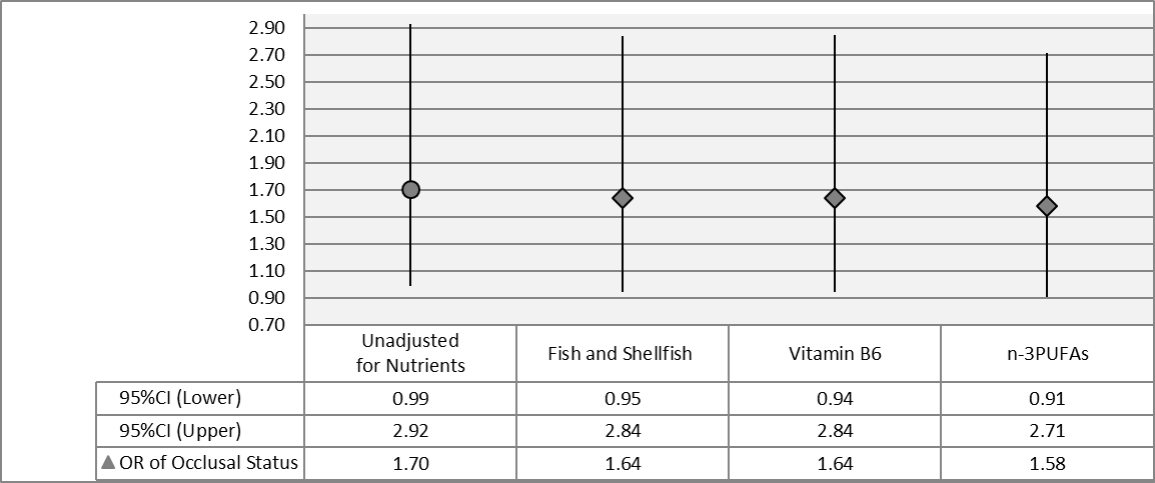
Odds ratio of occlusal status towards atherosclerosis assessing the effect of fish and shellfish, vitamin B6 and n-3PUFAs. CI: Confidence Interval, OR: Odds Ratio, n-3PUFAs: n-3 polyunsaturated fatty acid, Model*: adjusting for periodontal disease, gender, region of residence, smoking habits, drinking habits, education level, diabetes mellitus, hypertension, dyslipidemia, and hyperuricemia., Model**: Model* + (Fish & Shellfish), Model***: Model* + (Vitamin B6), Model****: Model* + (n-3PUFAs).

To assess whether these common related dietary factors have a mediating effect on the relationship between occlusal status and atherosclerosis, changes of odds ratio of occlusal status (Complete & Moderate groups vs. Severe group) on the prevalence of Atherosclerosis when adding the commonly related dietary factors were shown in Fig 1.

Odd ratio of occlusal status from the multiple logistic regression model with no adjustment for any dietary factor was 1.70 (95%CI: 0.99–2.92). However, odd ratio with adjustment for intake of fish and shellfish was reduced into 1.64 (95%CI: 0.95–2.84). Similarly, odds ratio with adjustment for vitamin B6 and n-3PUFAs were declined into 1.64 (95%CI: 0.94–2.84) and 1.58 (95%CI: 0.91–2.71), respectively. This means a statistical mediating effect of Fish and shellfish, vitamin B6 and n-3PUFAs within the relation between occlusal status and atherosclerosis.

## Discussion

This cross-sectional study showed an association between occlusal status and atherosclerosis, independent of periodontal condition. Collapsed occlusion in the posterior region was significantly related to the prevalence of atherosclerosis, as was severe periodontal disease. Besides, collapsed occlusion group tended to consume less of all key nutrients, and multivariable assessment showed intakes of green and yellow vegetables, fish and shellfish, vitamin B6, vitamin B12 and n-3 PUFAs were significantly declined in collapsed occlusion group, comparting to complete and moderate groups. Furthermore, fish and shellfish, vitamin B6 and n-3 PUFAs had the mediating effect within the relationship between occlusal status and atherosclerosis. Therefore, the null hypothesis was rejected. Within the limitation of a cross-sectional design, our findings may help to support the hypothesis of one pathway that poor intakes of specific nutrients resulting from decline in the occlusal support of posterior teeth could cause atherosclerosis, apart from periodontitis. This is the first report assessing the two pathways in the same population.

The relationship between periodontal disease and atherosclerosis has been the subject of growing research in recent decades [13], and a literature review reported a potential link between periodontal disease and atherosclerosis through the inflammatory effect of oral bacteria [15]. Another review concluded that there was consistent and strong epidemiologic evidence that periodontitis increases the risk of future cardiovascular disease [14]. We also found that severe periodontal disease was related to the prevalence of atherosclerosis. Therefore, the link between periodontitis and atherosclerosis very possibly exists.

A national diet and nutrition survey in the UK towards 955 older adults reported that the number of posterior tooth occlusion affected perceived chewing ability and food selection [1]. Japanese research groups also reported that loss of occlusion in the posterior region was related to lower consumption of vegetables or fish and shellfish [2,5], and our cohort had also a similar outcomes. The intakes of vegetables, fruits, fish and shellfish are regarded as the key for the prevention of cardiovascular disease, as is mentioned in the Japan Atherosclerosis Society (JAS) guidelines [25]. Vegetables and fruits are a primary dietary source of antioxidant vitamins such as vitamin A, E and C, dietary fiber and folic acid which reported on associations with reduced mortality from cardiovascular disease [6,7,27,30–32]. And fish and shellfish are rich in B-complex vitamins such as vitamin B6 and vitamin B12, and n-3PUFAs which were also reported to have a preventive efficacy of cardiovascular disease in several cohort studies [33–36]. In our cohort study, intake of fish and shellfish, and specifically consumption of vitamin B6 and n-3PUNFAs were significantly related to both of atherosclerosis and occlusal condition. Moreover, these indicated a mediating effect on the relationship between occlusal status and atherosclerosis (Fig 1). So that implies decreasing occlusal support of natural teeth might avoid eating fish and shellfish, and this lower intake of fish and shellfish containing of vitamin B6 and n-3PUNFAs would contribute to atherosclerotic plaque formation. As for vegetables and fruits, no significant relationship with the prevalence of atherosclerosis was observed in our study. And, although collapsed occlusal support group showed lower intake of green and yellow vegetable, intakes of other vegetable and fruit seemed to complement intakes of dietary fiber, vitamin A, vitamin C and folic acid.

As for other conventional risk factors in our study, the multivariable analysis showed that hypertension, dyslipidemia, and hyperuricemia were not significantly related to the prevalence of carotid atherosclerosis, whereas gender, smoking, educational level, and diabetes mellitus were related. The detailed mechanisms underlying the prevalence or absence of a significant relationship with these conventional risk factors cannot be explained based on this study. However, medications for hypertension, dyslipidemia, and hyperuricemia, which would indicate early detection and treatment at a routine medical check-up, could have influenced results. Furthermore, although the prevalence of hypertension and dyslipidemia was high in this study cohort compared with the general population, which would be expected to influence our results, the treatment status was not considered in the present study. In contrast, unmodifiable factors such as gender and smoking habits were shown to have a strong impact. Diabetes mellitus is generally hard to control, which explains why it is one of the main risk factors for atherosclerosis [37].

Because of this observational community-based study design, it cannot be denied that there might still be other hidden confounders’ effect or some sampling bias. However, our study was conducted by a complete enumeration survey method and participants were all of the septuagenarians (69–71 years old) recruited from both urban and rural regions and their dental and medical status was similar to the national data of Japan, indicating that our samples could be a representative of older adults in Japan. Since the general common risk factors of atherosclerosis were comprehensively covered to adjust those influence in this model and also the model was proved well fitted by global testing (p<0.001), it could be optimally designed. Nevertheless, to prove the causal association of our hypothetic pathways, the longitudinal data from our following survey are definitely required.

## Conclusions

In conclusion, within the limitations of the cross-sectional study design, which cannot prove causality, this study implied that poorer occlusal support of natural teeth was associated with the prevalence of carotid atherosclerosis. Keeping a good posterior occlusion of natural teeth would possibly be a supportive driving force to prevent cardiovascular or cerebrovascular accidents which are the most common causes of sudden death or disability of older adults worldwide. To establish affirmative evidence of the complex pathway between occlusal status and atherosclerosis via dietary intake, a prospective study is required.

## Supporting information

S1 File Minimal dataset. (XLSX)
